# Impedance Matching Antenna-Integrated High-Efficiency Energy Harvesting Circuit

**DOI:** 10.3390/s17081763

**Published:** 2017-08-01

**Authors:** Yuharu Shinki, Kyohei Shibata, Mohamed Mansour, Haruichi Kanaya

**Affiliations:** 1 Graduate School of Sciences and Electrical Engineering, Kyushu University, Fukuoka 819-0395, Japan; yuharu.917@gmail.com (Y.S.); 2ie16656p@s.kyushu-u.ac.jp (K.S.); 3IE16613T@s.kyushu-u.ac.jp (M.M.); 2 Electronics Research Institute, Dokki, Giza 12622, Egypt

**Keywords:** energy harvesting circuit, impedance matching, antenna, rectifier, radiofrequency

## Abstract

This paper describes the design of a high-efficiency energy harvesting circuit with an integrated antenna. The circuit is composed of series resonance and boost rectifier circuits for converting radio frequency power into boosted direct current (DC) voltage. The measured output DC voltage is 5.67 V for an input of 100 mV at 900 MHz. Antenna input impedance matching is optimized for greater efficiency and miniaturization. The measured efficiency of this antenna-integrated energy harvester is 60% for −4.85 dBm input power and a load resistance equal to 20 kΩ at 905 MHz.

## 1. Introduction

Battery-free and extremely low-power devices, such as wireless sensor nodes have attracted significant interest in the areas of information and communication research. Because of the energy limitations of the sensor battery, wireless sensors can only operate for a limited amount of time. It is possible to extend the lifetime of the wireless sensors by energy harvesting from natural sources, such as solar, wind and so on. However, energy from the environment such as through solar cells remains limited or is unstable in practice. On the other hand, radio frequency (RF) energy in wireless telecommunication systems represents a strong candidate for renewable energy harvesting. The study of cooperative transmission for simultaneous data and power in wireless networks involves an RF-based energy harvesting system. In this system, relay nodes harvest the ambient RF signal [1,2,3,4].

For circuit design, the development of a highly efficient RF energy harvesting circuit is required. A radio frequency identification (RFID) system operating in the 900-MHz band is suitable for RF energy harvesting because this frequency band has low consumption, high diffraction performance, and a 250-mW maximum transmitter power output. There is research on the topology of the RF energy harvesting circuit using transistors [5] or diodes [6,7,8]. In sensor node or Internet of things (IoT) devices, the structure of the diode is more simple and cost-efficient than the transistor. The main parts of energy harvesting circuits are diodes and capacitors, and the basic circuits are single-stage [6] and second-stage [7]. The maximum efficiency is 80% when input power is 10 dBm at a 900-MHz band [8]. However, because of the low RF power density, the stored energy is consumed by the parasitic resistance in the diodes and capacitors, and the efficiency of the circuit decreases remarkably. Therefore, a highly efficient circuit is needed even with low input power at less than 0 dBm.

In order to boost the low input voltage or low input power, the Cockcroft–Walton (CW) circuit [9] is utilized. In our previous research, a resonant circuit was constructed in front of the CW boost circuit to enhance the amplitude of the RF signal [10,11]. In a similar study, a simulation of high power conversion efficiency was reported using discrete parts [12]. However, for circuit design, high-frequency electromagnetic analysis is required to model the transmission line. Therefore, the impedance of the transmission lines and parasitic elements is considered through electromagnetic simulations. Moreover, both the circuit and antenna are optimized under the conditions of this study to maximize the power conversion efficiency.

Moreover, the antenna input impedance in high-frequency applications is 50 Ω to connect the transceiver or receiver circuit without reflection [5,6,7,8]. However, in order to further enhance the amplitude of the input voltage, it is necessary to optimize the antenna input impedance. In our previous reports, the design of the input impedance matching circuits and bandpass filters using a coplanar waveguide (CPW) transmission line is described [13,14,15,16]. Therefore, input impedance of the antenna is another design parameter for enhancing the input voltage.

In this paper, to improve input voltage, the input impedance of the antenna is optimized and an *LC* series resonance circuit is attached between the CW boost rectifier circuit and antenna. This circuit is a voltage amplification circuit and at the same time an impedance matching circuit. Moreover, the antenna and the *LC* and CW circuits are combined with size reduction.

The rest of the paper is organized as follows. In Section 2, the rectifier design will be discussed. Section 3, Section 4 and Section 5 describe the circuit measuring method by using the RF equipment, measured results of the DC voltage, and power efficiency, respectively. The antenna design, antenna measuring method, and measured results of the antenna are presented in Section 6, Section 7 and Section 8, respectively. In Section 9 and Section 10, the integration of the antenna and the circuit, and the measured results of the antenna with an impedance matching circuit are shown. Operation confirmation of the rectifier circuit integrated with an impedance matching antenna is discussed in Section 11. Finally, Section 12 summarizes the results of this work and draws conclusions.

## 2. Circuit Design

**Figure 1:**
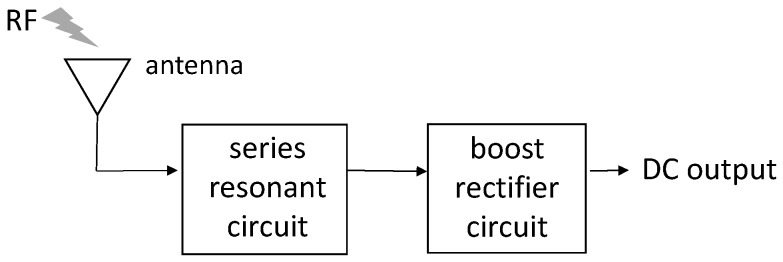
Block diagram of the energy harvesting circuit. RF: radio frequency.

**Figure 2:**
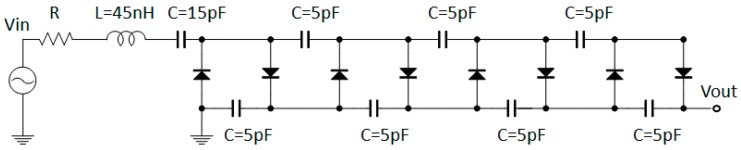
Schematic of the boost rectifier circuit. Vin: input voltage. Vout: output voltage. R: resistance. L: inductance. C: capacitance.

Figure 1 shows a block diagram of the proposed energy harvesting circuit with an antenna. The antenna with optimized input impedance, the series resonance circuit, and the boost rectifier circuits are all connected to each other. The weak input RF signal is amplified by the impedance matching circuit and series resonance circuit to obtain the DC output voltage. Figure 2 shows the circuit model of our proposed circuit. The CW boost rectifier circuit is composed of chip capacitors and diodes (HSMS-286K-G, Broadcom). In this study, the input power is very weak. Thus, a Schottky diode (SBD) is used owing to the excellent high-frequency operation and lowered threshold voltage compared to other diodes, such as the PN diode. Considering the internal structure of a diode, the CW circuit acts as the capacitor. The value of the *LC* series resonance circuit in front of the CW circuit compensates for the admittance of the CW circuit. The DC output of the proposed circuit is simulated by using the circuit simulator ADS (Keysight Tech., Santa Rosa, CA, USA).

**Figure 3:**
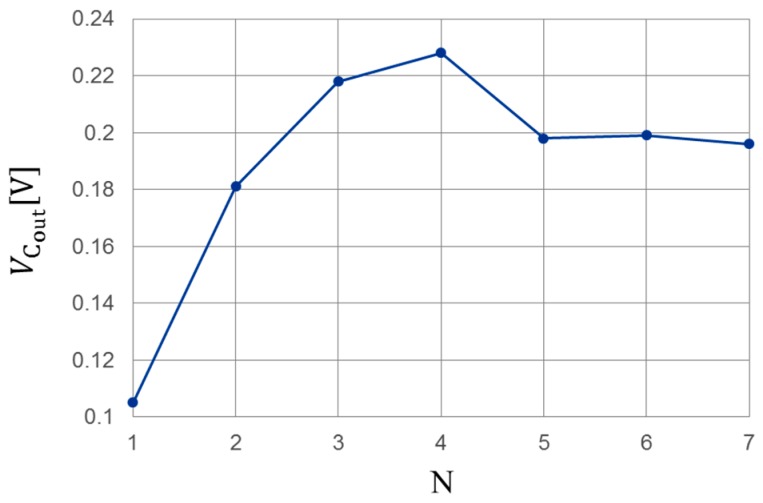
Simulation result of the number of stages in the Cockcroft–Walton (CW) circuit versus $V_{C_{\mathrm{in}}}$. $V_{C_{\mathrm{in}}}$: input voltage of CW circuit.

The output voltage of the CW circuit $V_{C_{\mathrm{out}}}$ is expressed as:

(1) $$V_{C_{\mathrm{out}}}=V_{C_{\mathrm{in}}}\times 2N$$

where $V_{C_{\mathrm{in}}}$ represents the input of the CW circuit, and *N* represents the number of capacitors and the diode unit structure. In the initial circuit design stage, the primary objective is to increase the output voltage, leading to efficiency. Therefore, a CW circuit which has high output power is obtained. Figure 3 shows the simulation results of the relationship between $V_{C_{\mathrm{out}}}$ and *N*. Here, $V_{C_{\mathrm{in}}}$ is 100 mV at 900 MHz, and the load resistance is an infinite value. From this result, Equation (1) is not satisfied because of the parasitic elements of the diodes. From Figure 3, $V_{C_{\mathrm{out}}}$ has the maximum at *N* = 4. Therefore, the optimum number of stages in this circuit is four.

To enhance the efficiency, a resistor-inductor-capacitor (*RLC*) series resonant circuit is connected in front of the CW circuit. The *RLC* series resonant circuit plays a voltage-amplifying circuit role. Namely, the input voltage is amplified by the quality factor (*Q* value).

**Figure 4:**
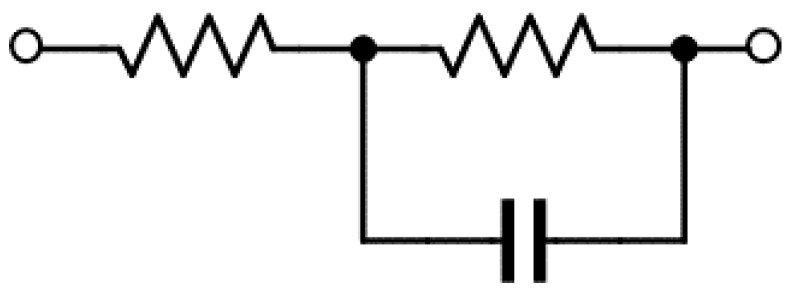
Schematic of an equivalent circuit of the diode.

The main factor behind low efficiency is the power consumption of the element internal resistance. Hence, the number of elements is reduced. The CW circuit can be regarded as a capacitor because a diode is represented, as shown in Figure 4. The capacitance value of this *RLC* circuit is the combined value of the resonance circuit and the matching capacitor. Thus, we succeed in reducing the number of elements. The value *R* represents the antenna input impedance. The *Q* value is described as:

(2) Q=1ωCR

**Figure 5:**
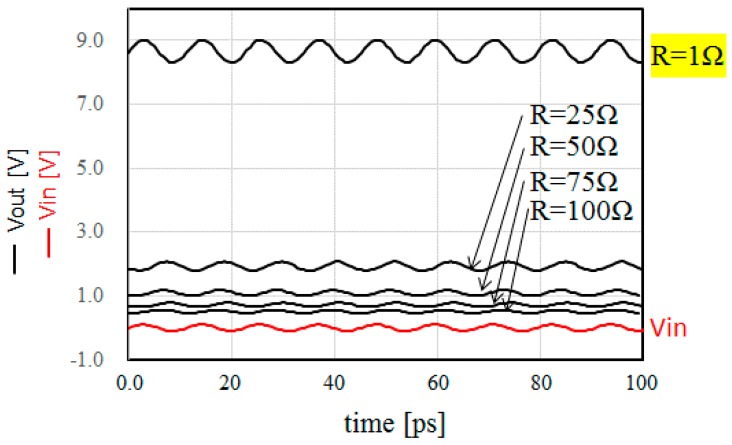
Transient simulation of the output voltage with different antenna impedances.

Considering Equation (2), the *Q* value is increased when *R* decreases. Thus, the input voltage is amplified and efficiency is also increased by changing the value of *R.* Figure 5 shows the time-domain simulation results in the steady-state of the DC voltage of the proposed circuit shown in Figure 2. As shown in Figure 5, when the antenna impedance *R* decreases, the DC output voltage increases. For the simulation, when *R* is less than 1 Ω, it is regarded as short. Thus, the minimum value of *R* is set as 1 Ω. For a 900-MHz application, the DC output voltage is 9.0 V at an input RF voltage of 100 mV and antenna impedance of 1 Ω.

## 3. Circuit-Measuring Method

**Figure 6:**
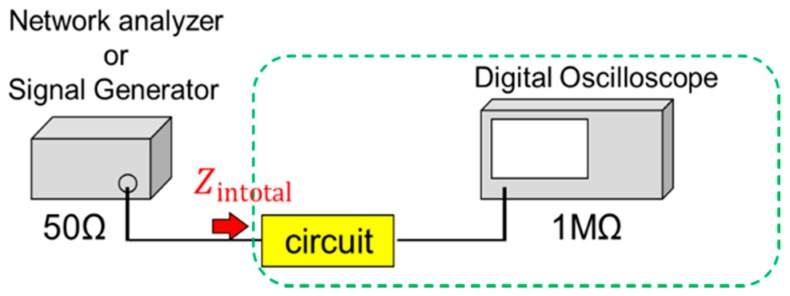
Simplified diagram of measurement.

Our proposed circuit cannot measure the performance directly because the impedance of the circuit is different from that of the measurement equipment (50 Ω). This leads to an impedance mismatch circuit. Therefore, we use a simulation to test the measurement method by considering attenuation in the coaxial cable and input circuit reflection. A 100-mV generator input voltage is then applied to the proposed circuit. The attenuation rate of the coaxial cable is measured at 10%. The reflection effect calculation method is shown as follows. To calculate this effect, it is necessary to measure the input impedance. Thus, the input impedance of the measurement system is measured by using a network analyser. Here, the measurement system of the proposed circuit, coaxial cable, and digital oscilloscope is indicated by a dotted circle in Figure 6.

At first, the reflection characteristics ($S_{11}$) of the measurement system are measured and the input impedance at resonant frequency $Z_{\mathrm{intotal}}$ is obtained by calculating Equations (3) and (4).

(3) $$Z_{11}=\frac{1+S_{11}}{1-S_{11}}\times Z_{0}$$

(4) $$Z_{\mathrm{in}}=Z_{11}-\frac{Z_{12}Z_{21}}{Z_{22}+Z_{L}}$$

where $Z_{0}$, $Z_{\mathrm{in}}$, and $Z_{L}$ represent port impedance, input impedance, and the load resistance, respectively. In this measurement, $Z_{L}$ is 1 MΩ because the input impedance of the oscilloscope is set as 1 MΩ to achieve the open-end state. Thus, from Equation (4), $Z_{\mathrm{in}}$ is approximately equal to $Z_{11}$. Therefore, it can be regarded as:

(5) $$Z_{\mathrm{intotal}}=\frac{1+S_{11}}{1-S_{11}}\times 50\;\left[\Omega \right]$$

The insertion loss $S_{21}$ due to an impedance mismatch is simulated when 50 Ω is connected with measuring equipment. From Equation (6), the input voltage of the generator is set for 100 mV of input.

(6) $$S_{21}\left[\mathrm{dB}\right]=10\log\frac{P_{\mathrm{output}}\left[W\right]}{P_{\mathrm{input}}\left[W\right]}$$

where $P_{\mathrm{input}}$ represents transmitted power from the generator and $P_{\mathrm{output}}$ represents power at the input of the circuit.

The circuit in Figure 2 is measured by the method shown above. However, the peak frequency is shifted to the lower frequency side. It is assumed to influence the transmission line and parasitic inductance component, because the circuit elements and transmission line are calculated as ideal. Therefore, the characteristic impedance and the transmission line loss are modelled by the electromagnetic simulator. Further, the circuit design is redesigned to match the peak frequency of 900 MHz.

**Figure 7:**
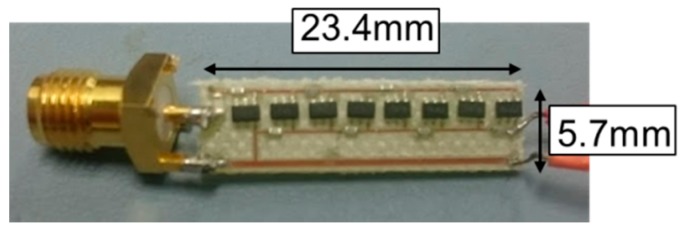
Photograph of the re-fabricated circuit.

Figure 7 shows a photograph of the redesigned boost rectifying circuit. The dimensions of this circuit are 23.4 mm × 5.7 mm. The substrate is FR4 with one side comprising a metal layer for combination with the antenna, as detailed in Section 6. The measured inductance value of the resonant circuit is 8.2 nH, and the measured $Z_{\mathrm{intotal}}$ is 11.2 Ω. From this result, the insertion loss $\left|S_{21}\right|$ is calculated to be 2.23 dB. The input value of the generator is then 273 mV when 100 mV of input voltage is applied to the circuit.

## 4. Circuit Measurement

**Figure 8:**
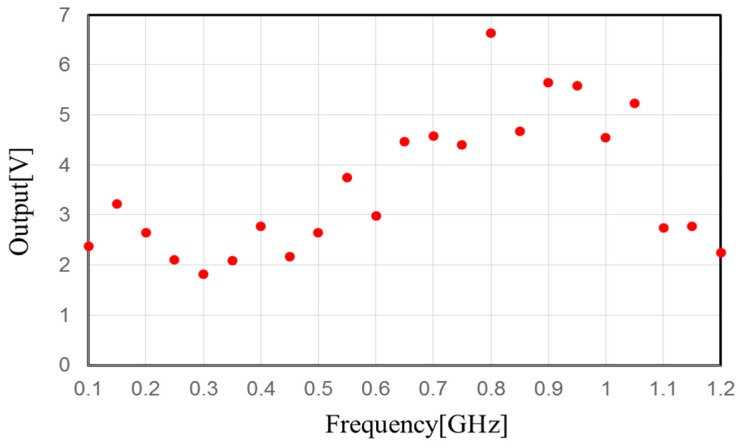
Measurement output voltage versus input RF signal.

Figure 8 shows the measurement results of the output DC voltage at a load resistance equal to 1 MΩ with different input frequencies. As shown in Figure 8, the DC output voltage is boosted for 100 mV of input. The output voltage is highly boosted for 800 MHz to 1.05 GHz and the central frequency of the higher-boosted voltage frequency is around 900 MHz. Therefore, the DC output voltage of this circuit is 5.67 V for a 900-MHz, 100-mV alternating current (AC) input.

## 5. Power-Conversion Efficiency Measurement

**Figure 9:**
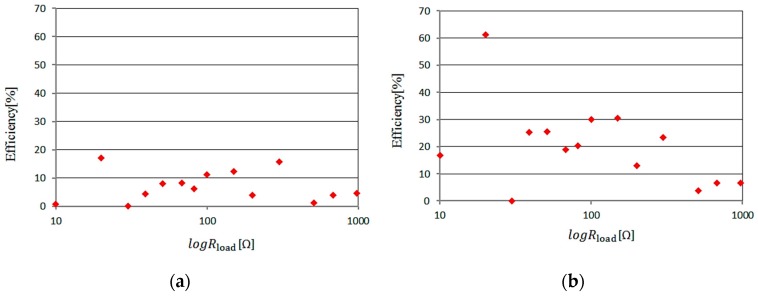
Measurement output voltage versus input RF signal. (**a**) Input voltage is 50 mV (**b**) input voltage 100 mV.

In this section, the maximum conversion efficiency is measured by changing the value of load resistance. The power conversion efficiency is represented as:

(7) $$\eta_{0}\left[\%\right]=100\times \frac{DC\;output\;power\;\left[W\right]}{incident\;RF\;power\;\left[W\right]}$$

where the incident RF power represents the input power into the measurement system, and the DC output power represents the output voltage measured at the load resistance. Figure 9 shows the measurement results. Two cases of the input power into the measurement system (50 mV and 100 mV) are shown as examples. When the value of the load resistance is 20 kΩ, the maximum efficiency is 17.2% and 61.2% for the 50 and 100 mV inputs, respectively. Therefore, the optimal value of the load resistance is 20 kΩ.

## 6. Antenna Design

**Figure 10:**
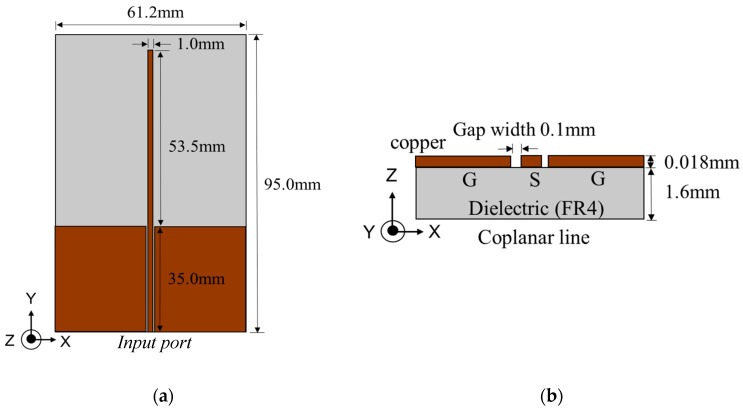
Layout of the proposed antenna. (**a**) layout, (**b**) cross sectional view.

The antenna requirements are an input impedance of 1 Ω and a unidirectional radiation. Figure 10 shows the layout of the proposed antenna. This antenna is a quarter wavelength monopole antenna with a CPW transmission feed line and a one-sided metal layer. Considering the current flow of the monopole antenna, it is open-edged and short in the port. Moreover, the radiation pattern of the monopole antenna is unidirectional.

Basically, the guided wavelength $\lambda^{\prime }$ is expressed as:

(8) $$\lambda^{\prime }=\frac{\lambda }{\sqrt{\varepsilon_{r}}}$$

where λ and $\varepsilon_{r}$ represent the target wavelength (frequency) and the dielectric constant, respectively. Considering the structure of the CPW, it is necessary to calculate the effect of air because electromagnetic waves are transmitted in both the air ($\varepsilon_{r}=1)$ and the dielectric. The guided wavelength considering this is represented as follows [17]:

(9) $$\lambda^{\prime }=\frac{\lambda }{\sqrt{\frac{\varepsilon_{r}+1}{2}}}$$

Equation (9) holds for the case where the dielectric has enough thickness. However, in this study, the thickness is limited. Therefore, the guide wavelength of the proposed antenna is calculated by using an electromagnetic simulator (HFSS by ANSYS). The substrate has a dielectric constant of $\varepsilon_{r}=4.4$ and tan δ = 0.02. The thicknesses of the copper layer and substrate are 18 μm and 1.6 mm, respectively.

**Figure 11:**
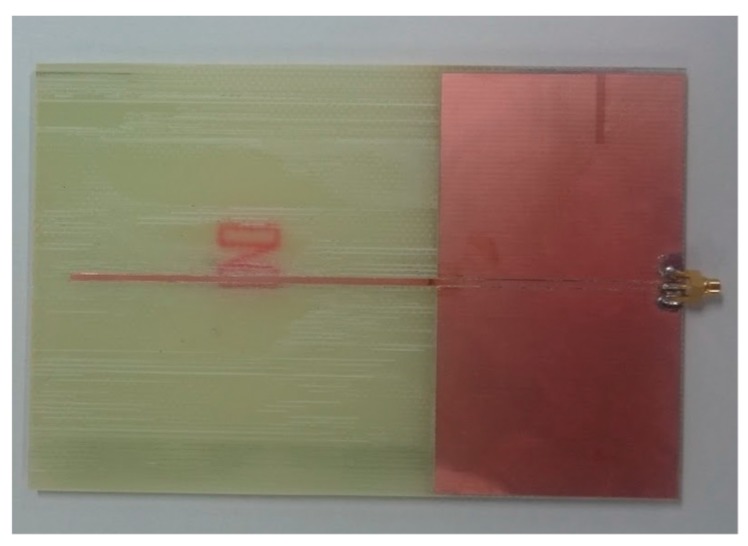
Photograph of the fabricated antenna.

Figure 11 shows a photograph of the fabricated antenna. Input impedance is optimized for the size of the line and space. Because the minimum line and width of our printed circuit board maker is 0.1 mm, the minimum impedance is 4.4 Ω instead of 1 Ω. Therefore, we succeeded in designing the antenna with a low input impedance.

## 7. Antenna Impedance Measuring Method

It is impossible to measure the frequency characteristics of the proposed antenna directly because the input impedance of this antenna differs from 50 Ω. Therefore, we represent the measurement method using a simulation. Antenna measurement conditions are shown as follows:

Case A: (coaxial cable and connector, port, antenna) = (50 Ω, 50 Ω, 4.4 Ω)
Case B: (coaxial cable and connector, port, antenna) = (removed, 4.4 Ω, 4.4 Ω)

Case A represents the actual measurement condition and Case B represents the ideal condition. Case B can measure the characteristics of this antenna without reflection. In this study, the coaxial cable and connector are not necessary because the antenna and circuit are fabricated on the same substrate. Therefore, the component of the coaxial cable and connector is “removed”.

**Figure 12:**
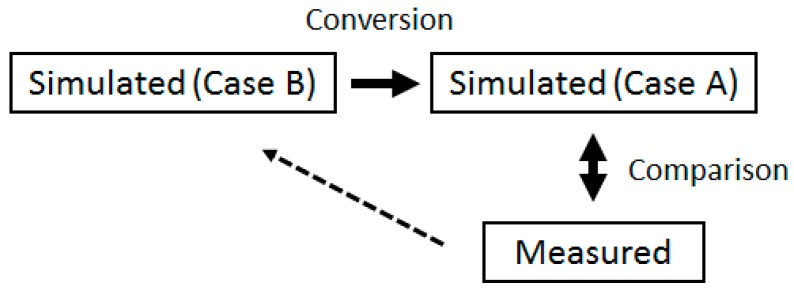
Block diagram of the measuring method of the proposed antenna.

Figure 12 shows the block diagram of the measuring method. The calculation results of Case B are converted to Case A, and then compared to the measurement results. If they correspond to each other, the characteristics of the fabricated antenna and Case B are equal. The conversion of Case B to Case A means calculating the characteristics for changing measurement conditions only. First, the characteristics of S_11_ and the radiation pattern are calculated under Case A and B. The simulation results are then compared to the measured results.

## 8. Antenna Measurement

**Figure 13:**
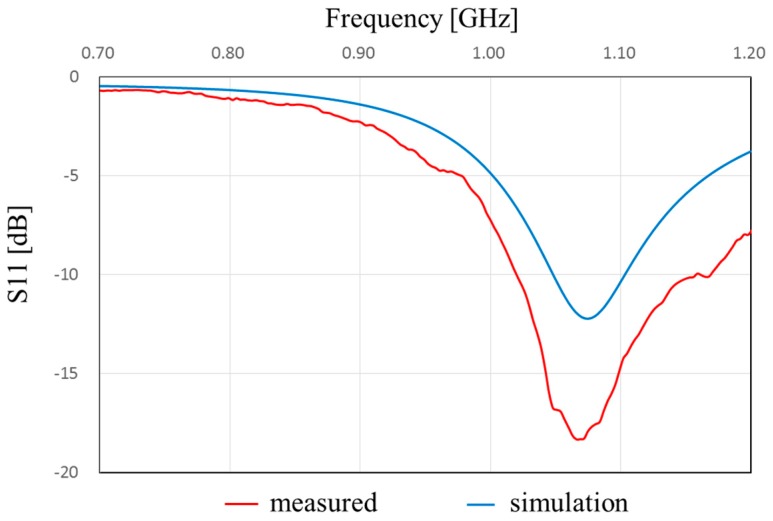
Frequency characteristics of S_11_.of the proposed antenna in Case A.

**Figure 14:**
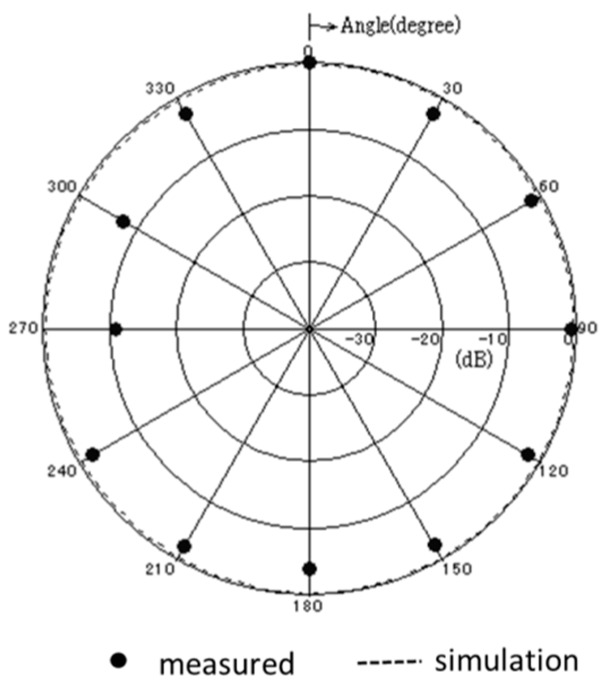
Measured and simulated radiation pattern of the proposed antenna.

**Figure 15:**
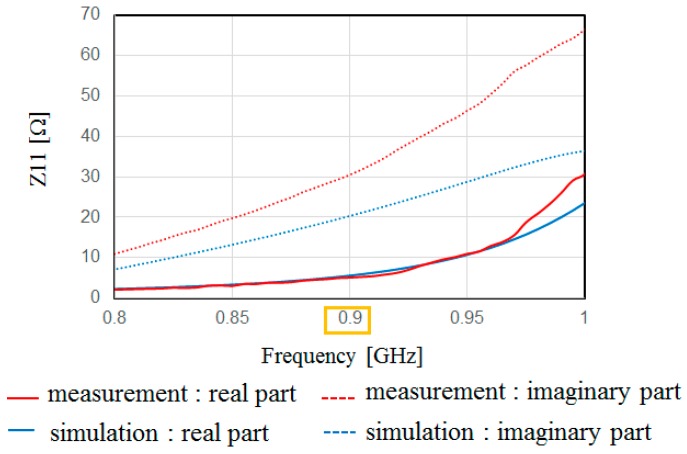
Measured and simulated frequency characteristics of Z_11_ calculated from *s*-parameters.

Figure 13 shows the measured and simulated Case A characteristics of S_11_. The measured peak frequency corresponds to the simulated results. The value of S_11_ is slightly shifted, and the impedance approaches 50 Ω because of the soldering effect of the connector. Figure 14 shows the measured and simulated radiation pattern of the *x*-*z* plane. From this result, it is confirmed that the fabricated antenna has unidirectional radiation. Figure 15 shows the measured and simulated (Case A) impedance Z_11_ calculated from S_11_. The real parts of the measured results are approximately equal to those of simulated Case A at 900 MHz. On the other hand, the measured imaginary part is slightly higher than that of the simulated results because of the error from the parasitic elements of soldering. Therefore, we can conclude that the fabricated antenna has the characteristics of Case B and it successfully attains the intended characteristics of the fabricated antenna.

## 9. Integration of the Antenna and the Circuit

**Figure 16:**
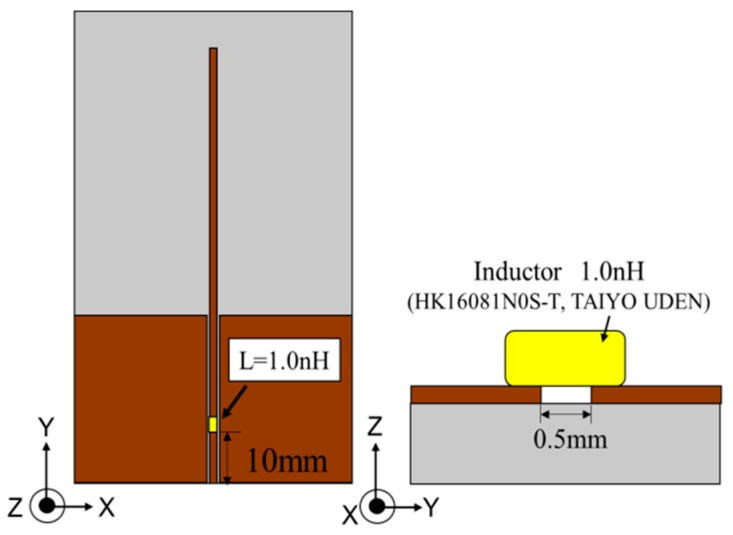
The top view (**right**) and side view (**left**) of the antenna with an impedance matching circuit.

In order to integrate the antenna and the circuit, it is necessary to match the impedance because the impedance of the antenna is different from the circuit. Therefore, we designed the impedance matching circuit. The circuit is to be integrated into the antenna for the convenience of the design. In the circuit, $Z_{\mathrm{intotal}}$ is 13.2 + *j*0.0 (Ω) at the 905 MHz measurement resonant frequency. Thus, the impedance matching circuit is designed for 905 MHz. Figure 16 shows the antenna design with the impedance-matched circuit. A 1.0-nH inductance is set at a 10-mm point from the input port.

**Figure 17:**
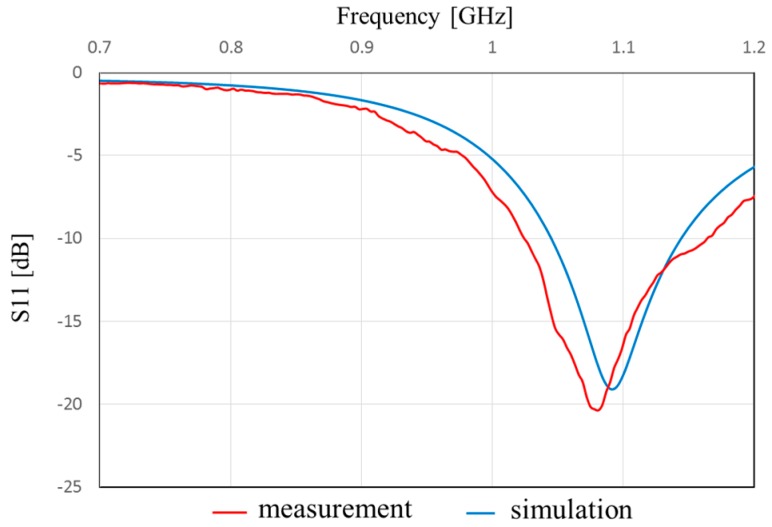
Frequency characteristics of S_11_ of the antenna with an impedance matching circuit (Figure 16).

Figure 17 shows the simulation result of the S_11_. From this result, S_11_ is less than −10 dB. Namely, the transmission rate is more than 90% from 875 MHz to 915 MHz. Therefore, impedance matching between the antenna and the circuit is confirmed at 905 MHz.

## 10. Measurement of the Antenna with Impedance Matching Circuit

We fabricate the antenna as per Figure 16 and measure it the same way as mentioned in Section 7. Antenna conditions are provided below:

- Case C: (coaxial cable and connector, port) = (50 Ω, 50 Ω)
- Case D: (coaxial cable and connector, port) = (removed, 13.2 + *j*0 Ω)

**Figure 18:**
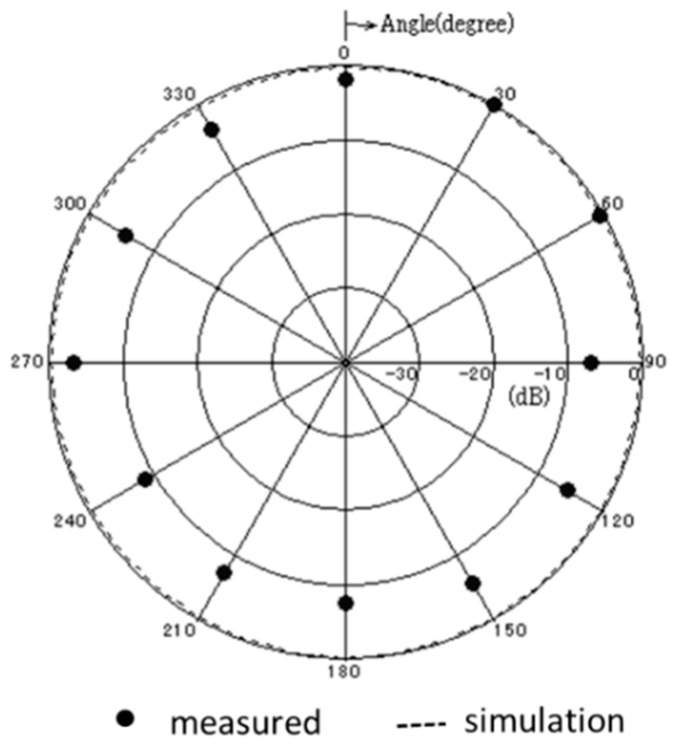
Measured and simulated radiation pattern of the antenna with an impedance matching circuit.

**Figure 19:**
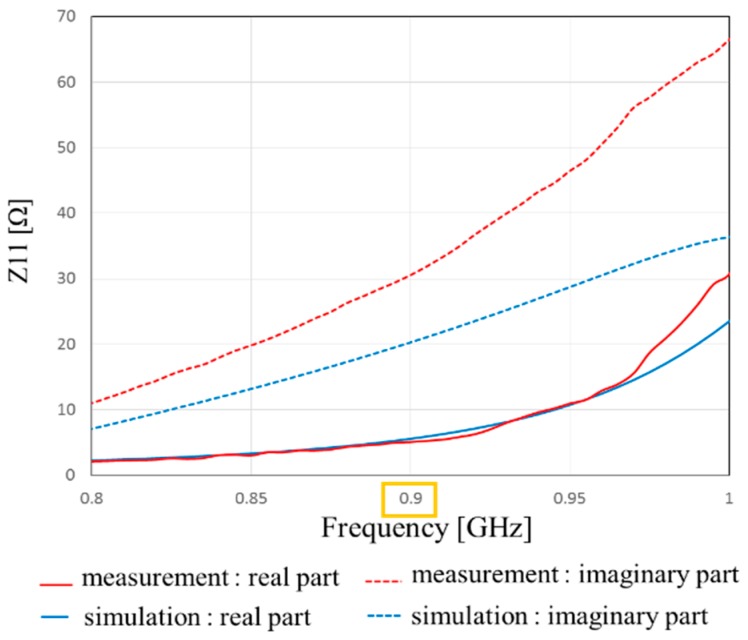
Frequency characteristics of Z_11_ of the proposed antenna with impedance matching circuit calculated from s-parameters.

Figure 17 shows the measured and simulated Case C characteristics of S_11_. Figure 18 shows the measured and simulated radiation pattern of the x-z plane. From this result, it is confirmed that the fabricated antenna has unidirectional radiation. Therefore, we can fabricate the antenna with the intended circuit characteristics. Figure 19 shows the measured and simulated Case C impedance Z_11_ calculated from S_11_. From this result, the real part of the measured result is approximately equal to that of the simulated Case C at 900 MHz. On the other hand, the measured imaginary part is slightly higher than that of the simulated result because of the error from the parasitic elements of soldering.

## 11. Operation Confirmation of the Circuit Integrated with an Impedance Matching Antenna

**Figure 20:**
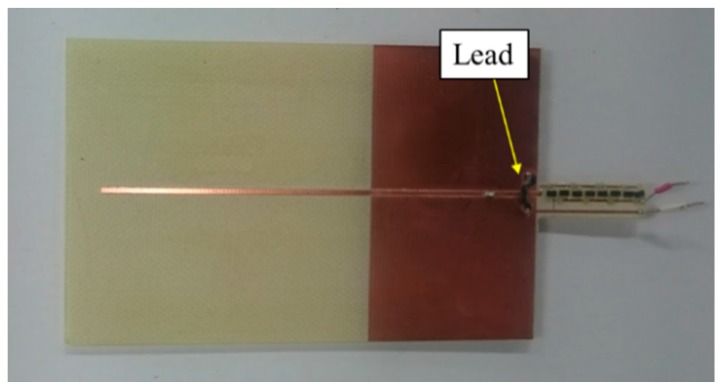
Photograph of the fabricated antenna with the impedance matching antenna.

**Figure 21:**
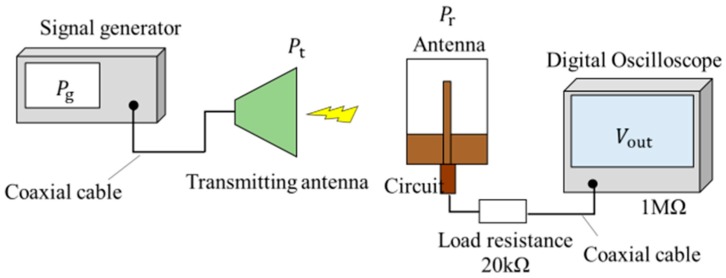
Measuring flow of the operation confirmation. *P_g_*: output power from a signal generator. *P_t_*: transmitting power. *P_r_*: receiving power.

Figure 20 shows a photograph of the fabricated antenna and circuit on the same substrate. A ground line of the circuit is connected to one ground of the antenna. Thus, grounds are connected by using a lead line. The dimensions are 61.2 mm × 118.4 mm. Figure 21 shows a measuring flow of operation confirmation. First, input power is transmitted to the transmitting antenna from the signal generator. Then, the output voltage $V_{\mathrm{out}}$ of the circuit with the antenna is measured by the digital oscilloscope. The circuit with the antenna is measured on the wave absorber to reduce the influence of reflection. The value of the load resistance is 20 kΩ, as mentioned in Section 5, and that of the oscilloscope is 1 MΩ.

In this measurement, power conversion efficiency is measured by changing the input power. Namely, it is equal to the distance difference between the transmitting and receiving antennas. The measured frequency is 905 MHz because the resonant frequency of the circuit with the antenna is 905 MHz. The power conversion efficiency is represented as Equation (7). Considering these measured conditions, the efficiency is represented as follows:

(10) $$\eta_{0}\;\left[\%\right]=100\times \frac{{{V^{\prime }}_{out}}^{2}/\left(20\times 10^{3}\right)\left[W\right]}{P_{r}\left[W\right]}$$

where $P_{r}$ represents the receiving power and ${V^{\prime }}_{out}$ represents the output voltage that is considerably attenuated with a coaxial cable. $P_{r}$ cannot be measured directly due to the design. Thus, $P_{r}$ is calculated by using the Friis transmission equation. This equation is represented as:

(11) $$P_{r}=G_{r}G_{t}P_{t}{\left(\frac{\lambda }{4\pi r}\right)}^{2}$$

where $G_{r}\;\mathrm{and}\;G_{t}$ are the gains of the receiving antenna and transmitting antenna, respectively. $P_{t}$ represents the transmitting power. r represents the distance between the transmitting and receiving antennas. λ represents the wavelength frequency. In this measurement, the conditions are $G_{r}=1$, $G_{t}=2.2$, r = 0.55 m, and λ = 0.331 m.

Table 1 shows the measurement results. This output voltage is the average output of the three measurements.

## 12. Conclusions

In this study, a high-efficiency energy harvester was developed. For the boost rectifier circuit, the DC output voltage is 5.67 V for input voltage of 100 mV AC and 900 MHz. A low-input impedance antenna was successfully developed. In addition, we formulated the antenna measurement method, integrated the circuit, and impedance-matched the antenna. This final efficiency is 60% for a load resistance equal to 20 kΩ when the input power is −4.85 dBm.

## Figures and Tables

**Table 1:** Performance comparison of the efficiency at 900 MHz band energy harvesting circuit.

*P_r_* [dBm]	*V’_out_* [V]	Efficiency *η*_0_ [%]	*η*_0_ [%] [8] *
−2.85	2.67	68.7	52.5
−4.85	1.88	60.0	46.3
−7.85	1.05	38.8	32.5

* At 940 MHz. Estimated from Figure 7 in [8].
